# Community structure and timing of sexual activity among adolescent girls in Nigeria

**DOI:** 10.1371/journal.pone.0269168

**Published:** 2022-07-27

**Authors:** Oluwaseyi Dolapo Somefun, Emmanuel Olamijuwon

**Affiliations:** 1 UKRI GCRF Accelerating Achievement for Africa’s Adolescents, School of Public Health, University of the Western Cape, Cape Town, South Africa; 2 School of Primary Care, Population Sciences and Medical Education, Faculty of Medicine, University of Southampton, England, United Kingdom; 3 School of Geography and Sustainable Development, University of St Andrews, Scotland, United Kingdom; 4 Demography and Population Studies Programme, Schools of Public Health and Social Sciences, Faculties of Health Sciences and Humanities, University of the Witwatersrand, Witwatersrand, South Africa; University of Salamanca, SPAIN

## Abstract

Studies have linked the timing of sexual debut to unplanned pregnancies and sexually transmissible infections, including HIV. Current understandings of sexual debut among Nigerian adolescents focused on the roles of individual and familial characteristics. We leveraged the 2018 Nigeria Demographic and Health Survey data to examine how community features like affluence, ethnic diversity, and women empowerment may be associated with the timing of sexual debut among adolescent girls. The sample comprised 7449 adolescent girls who were usual residents in 6,505 households and 1,352 clusters or communities. Statistical associations between community characteristics and the onset of sexual debut were assessed using a two-level mixed-effects parametric survival model with Weibull distribution. We found that community affluence [aHR:0.43, 95%CI: 0.30–0.62] and community ethnic diversity [aHR: 0.63, 95%CI: 0.42–0.94] are associated with a lower hazard of sexual debut among adolescent girls. We also observed that women that married within the observation period had an earlier sexual initiation than those who were unmarried. The results disaggregated by marital status further shows that higher community level of women’s employment [aHR: 2.45, 95%CI: 1.38–4.38] and women’s education [aHR:1.85, 95%CI: 1.03–3.33] were associated with a higher hazard of sexual debut among unmarried adolescent girls but not married adolescent girls. Higher community affluence [aHR:0.40, 95%CI: 0.27–0.60] was also associated with a lower hazard of sexual debut among unmarried adolescent girls but not married adolescent girls. Our results illuminate the associated factors of the timing of sexual debut among adolescent girls that moves beyond individual characteristics to community characteristics.

## Introduction

The timing of sexual intercourse has considerable significance for young adults’ life course [1, 2]. Young adults, particularly in African countries, often do not have access to comprehensive sexuality education that could enhance their agency in romantic relationships and efficacy for contraceptive use. As a result, early sexual debut among this group has been linked to several negative outcomes, which include low educational attainment [3], unplanned pregnancy [4, 5], sexually transmitted infections including HIV [6, 7], and higher adolescent fertility rate [8, 9]. These risks altogether make the timing of sexual intercourse a public health concern and the primary focus of research into the sexual behaviour of young people.

Unplanned pregnancy, especially outside marriage, is usually seen as a desecration of moral values in Nigeria [10]. Stigmatization due to unplanned pregnancy affects young adults in different ways. These include their access to reproductive health facilities [11], leading to another unplanned pregnancy [12], and low educational attainment following their ostracization at learning institutions [13] and other social networks. Interestingly, those perpetrating such stigma consider it a protective factor against unintended pregnancy among young adults, enabling this stigma to linger long in these settings [10]. Beyond the stigma, unplanned sex among young adults may result in higher rates of adolescent pregnancies due to a lack of capacity to negotiate condom use.

However, social norms are changing [14] due to globalization and the diffusion of ideas. This change also leads to a breakdown of traditional norms due to increased social learning (through social media), noted as an essential mechanism through which new behaviours evolve [15, 16]. Knowing how community factors influence the timing of sexual debut in the context of these changes becomes imperative, especially the relationship between a neighbourhood normative climate [17], its structure, and the timing of sexual debut in Nigeria. A holistic approach to understanding the sexual behaviour of young adults is crucial for policy planners to evaluate and sustain the existing sexual and reproductive health services available to them.

### Theoretical framework and prior work

Several studies have examined the timing of sexual debut among young adults [18–20]. For instance, examining sexual debut among 15-year-old in-school adolescents in eight African countries, Peltzer [21] reported that 27.3% had experienced sexual debut before age 15, 38.1% among boys and 15.8% among girls. Another study exploring the timing and circumstances of first sex among female and male youth in Nigeria, Kenya, and Senegal found that more than half of the youth aged 20–24 in Kenya had engaged in premarital sexual activity [22]. The results also showed that transition to premarital first sex was more common among Kenyan youth followed by Nigerian youth.

However, country-level results are varied. For instance, in Ethiopia, a school-based cross-sectional study found that 19% of the students had early sexual debut [23], while results from another cross-sectional study of adolescents aged 16–19 in Tanzania revealed that 5% had sex before their 15th birthday [24]. Findings based on data from five consecutive National HIV & AIDS and Reproductive Health Surveys (NARHS 2003, 2005, 2007 and 2012) showed a significant decline in the proportion of young women (16.6 per cent to 9.2 per cent) and men (6.0 per cent to 4.5 per cent) reporting sexual debut before age 15 [25].

Similar results emerged from other studies conducted in Nigeria [26, 27]. Specifically, these studies concluded that factors such as age, sex and beliefs operate at the individual level while some determinants operate at the family and the broader socioeconomic environment levels [28, 29]. More recently, Guilkey & Speizer [30] found that that community beliefs and attitudes were associated postpartum contraceptive use among young adults in Nigeria. Despite the amount of research carried out to explore individual risk factors associated with the timing of sexual debut, fewer studies have explored the role of the community [28, 31], especially in Nigeria. For example, Tenkorang and Maticka-Tyndale [28] found that men and women had a lower likelihood of early sexual debut if they resided in a community with high HIV knowledge and communities that focused on abstinence-only messages as a prevention strategy. This study shows the association between neighbourhood normative climate and youth sexual behaviour.

In this study, we leveraged the ecological model to explain the timing of sexual debut among adolescents in Nigeria. The ecological model posits a relationship between individuals and the environment in which they live [32]. Bronfenbrenner recognized specific systems that could influence an individual—the micro-, meso-, exo- and macro- systems. The microsystem explains how characteristics in the individual’s environment, like the family and social networks, influences youth outcomes. The mesosystem refers to the relationships between the hypothesized microsystem factors. The exosystem refers to external dynamics in the larger society where the individual resides, although the individual does not have an active role within the larger society. Contributing factors would be community socioeconomic status and community-level fertility rates. The macrosystem refers to traditional norms and values that influence the other systems. In this study, the microsystem refers to youth characteristics. The exosystem, on the other hand, refers to the community characteristics. Factors at the individual level include age, religion, region, and education of individuals.

As part of this growing body of literature on community norms and youth behaviours, the effect of community norms on youth behaviours may be direct or operate through some other mechanisms at the family or individual level [33]. It is also known that these levels are interrelated. For instance, family structure and socioeconomic characteristics at the family level could influence the place of residence, and the place of residence could also influence the choice of peers. Likewise, community socioeconomic crises such as the proportion of highly educated adult women may also influence the onset of premarital sexual debut among adolescents. This observation builds on the view that a community with more educated or working women may be more likely to educate youth about the risk of early sexual debut [34]. Youths from communities with high socioeconomic status may have more resources available to them, including financial, which may influence their timing of sexual debut when compared to their counterparts from low socioeconomic backgrounds [35].

Based on this background, we examined three distinct community features (community level of affluence, community level of women’s empowerment and community diversity measured as ethnicity) and how they may be associated with adolescents’ timing of sexual debut. These community-level variables measure social mobility, described in other studies [34, 36]. Specifically, we hypothesized that:

1. H_1_: Adolescents in more affluent communities will differ in their onset of sexual debut compared to adolescents in less affluent communities.
2. H_1_: Adolescents in communities with a high proportion of adult women working for cash or cash-kind will differ in their onset of sexual debut compared to adolescents in communities with a low proportion of adult women working for cash or cash-kind.
3. H_1_: Adolescents in communities with a high ethnic diversity will differ in their onset of sexual debut during adolescence compared to adolescents in communities with low high ethnic diversity.

Exploring how these factors are associated with the timing of sexual debut among adolescents in Nigeria could help sustain strategies that support delayed timing of sexual intercourse. It can also help develop context-specific interventions focusing on sexually active adolescents. In addition, evaluating the association between community characteristics and the timing of sexual debut could shed light on the possible mechanisms by which neighbourhoods shape youth sexual behaviour.

## Materials and methods

### Data

We obtained data from the women’s module of the most recent Demographic and Health Survey (NDHS) conducted in 2018. The NDHS is a large-scale national survey that collects demographic and health information on various demographic and health indicators, including individual characteristics, marriage and sexual activity, knowledge and use of family planning services, and HIV/AIDS-related knowledge, attitudes and behaviour. The survey’s rich information on the sexual and reproductive activity of adolescent girls and adult women make it a valuable resource for this study.

### Study sample

The dataset comprised women in the reproductive age group 15–49 years. Since we aim to provide a better understanding of sexual debut among adolescent girls, we focus on a subset of the data from 8,402 adolescent girls aged 19 years or younger at the time of data collection. Based on our research’s peculiar focus on community characteristics and to account for the temporality of events, we further excluded 953 adolescent girls who were visiting the selected households or who had their first sexual activity before moving into the community where the interview took place. This exclusion also helps us ensure that the adolescent girls included were exposed to sexual activity in the community where they were interviewed. The final sample of adolescents who met the study criteria was 7,449 adolescent girls aged 15–19 years who were usual residents in 6,505 households nested in 1,352 clusters or communities.‬‬‬‬‬‬‬‬‬

### Measures

#### Outcome variable

The outcome variable for this study is the timing of first sexual activity among adolescent girls in Nigeria. We measured this in years from the year of adolescents’ birth to the year of first sex. Using the information on adolescent girls’ reported sexual activity, we created a status variable to indicate whether adolescent girls have had sex or not. We classified adolescent girls as sexually experienced (1) if they reported having had sex and not sexually experienced (0) if otherwise. By adopting an event-history modelling approach, we used the information on age at first sex for adolescent girls who reported that they have ever had sex. In contrast, adolescent girls who reported never having sex were censored at their age during the interview. The final measurement variable ranged from 8 (onset of sexual activity in the sample) to 19 years (end of adolescence).

#### Community-level variables

The community-level variables in this study include community level of affluence, community level of women’s empowerment and community ethnic diversity. We considered each of the primary sampling units in the datasets as communities for this study. As a result, all the community-level characteristics were created by aggregating individual-level characteristics to the level of the primary sampling unit. Similar consideration has been extensively adopted in analyzing community effects on sexual and reproductive health outcomes and behaviours, mainly using the Demographic and Health Survey [37, 38]. This approach enables us to critically appraise the associations between community-level characteristics and the timing of sexual debut among adolescents.

The community-level women’s empowerment was measured using two proxy measures–education and employment. These measures were generated from self-reported responses of adult women aged 25–49 years who were usual residents in the selected household and community. A similar approach was adopted to study adolescent sexual behaviour in the United States [39]. The *community level of women’s educational status* was defined as the weighted proportion of adult women aged 25 years and above with tertiary or higher education in each sampling unit. *The community level of women’s employment status* was measured as the weighted proportion of adult women aged 25 years and above who reported being currently working at the time of data collection and were working for cash or cash-kind in each sampling unit.

*Each sampling unit measured the community level of affluence* as the weighted proportion of households in the richest wealth quintile (households in the top 40% of the wealth index). *The community level of ethnic diversity* was measured as the weighted proportion of adolescents from major ethnic groups (Yoruba, Igbo, Hausa, Fulani, and Ijaw) residing in each sampling unit. Weighted proportions for all the community characteristics were estimated using the *asgen* function in STATA to adjust for the complex design of the sample [40].

#### Covariates

In order to illuminate the relationship between the community variables and the timing of first sexual activity among adolescent girls, our analysis includes several individual-level socio-demographic characteristics of adolescent girls. These variables include adolescent girls’ highest level of educational attainment, marital status, place of residence, ethnicity, and religious affiliation. The selection of these variables was based on prior evidence of their associations with early sexual debut [24, 26, 41].

Adolescent girls’ highest level of educational attainment was classified as less than secondary (1) for adolescents who reported to have never completed primary or have no formal education and secondary or higher education (2) for those with secondary or tertiary education. Marital status was categorized as never married (1) for adolescent girls who have never been married and (0) for adolescent girls who have ever been married, including those who are currently married, living with a partner, divorced, separated or widowed. The place of residence was also an individual level characteristic classified as urban (1) and rural (2). Region of residence describes the geo-political zone in which the adolescent girls reside. Response categories for the variable were classified into North Central (1), North East (2), North West (3), South East (4), South South (5), and South West (6). Ethnicity was a categorical measure of the ethnic group to which adolescents belong. Response to the variable was categorized to reflect the country’s major ethnic groups (Yoruba, Igbo, Hausa, Fulani, and Ijaws). We further classified all other minority ethnic groups as others. Religious affiliation was categorized into Christians (1) comprising all Christian denominations including Pentecostal, Catholic, protestant and other Christian denominations, Muslim, and others (3).

### Statistical analysis

We used frequency and percentage distributions to describe the characteristics of adolescent girls in the sample both at the individual and community levels. For this analysis, frequency distributions were unweighted, while percentage distributions were weighted to account for the complex design of the survey. Mean and standard deviations were computed for all continuous exposure variables, more precisely, the community level characteristics. Stata software 14.1 [42] was used for all analyses.

Kaplan–Meier curve was used to estimate the survival probability of sexual debut by 19 years among adolescent girls in the sample. We also compared survival probability between adolescents in highly vs less affluent; highly diverse vs less diverse; high vs low proportion of educated adult women, and high vs low proportion of women working for cash communities. Since these variables were continuous, we dichotomized all community level characteristics as low (proportion < 0.50) and high (proportion ≥ 0.50) for this comparison. Log-rank test was used to delineate statistically significant differences between the groups for all community characteristics.

Finally, to answer our main research question of how community-level characteristics are associated with the timing of first sex, we fitted two multilevel mixed-effects parametric survival models with Weibull distribution using the *mestreg* function. *mestreg* allows us to combine the modelling of the hierarchical nature of the data with parametric analysis timing of sexual debut. The first model comprised all community-level characteristics and the timing of sexual debut, while the second model comprised the first model and individual-level socio-demographic characteristics of adolescent girls. Considering that the timing of sexual debut is likely to differ between unmarried and married adolescents, we further stratified our main models by marital status. Precisely, community-level characteristics could increase the likelihood of premarital sexual activity. At the same time, for married adolescent girls, community-level characteristics likely motivate girls to marry at earlier ages and subsequently sexual initiation in adolescence (early marital sexual debut). All the models were also weighted to account for the complex design of the survey.

Variations within the neighbourhood (random effects) were assessed using interclass correlation (ICC) and proportional change in variance (PCV). The ICC shows the variation in the timing of sexual debut among adolescent girls and young women due to neighbourhood characteristics. The PCV, on the other hand, measures the total variation attributed by individual-level variables and neighbourhood-level variables in the final/full model. The PCV was calculated as the difference between the variance of the null model and each of the models divided by the variance of the null model.

Hazard ratios, 95% confidence intervals and p-values were reported. All data analyses were weighted at the individual level using survey weights to adjust for all analyses’ differential probabilities of selection and nonresponse.

## Results

### Descriptive profile of sample

Table 1 summarizes the descriptive profile of adolescent girls in the sample. About two-thirds (66%%) of the adolescent girls have attained secondary or higher levels of education. More than half of the adolescents (53%) reside in rural areas, and about 64% of the adolescents reside in the country’s northern region. The majority of the girls have never been married, while about 49% are Christian. About 17% are Yoruba, while 16% are Igbo and 27% are Hausa. Table 2 presents the mean and median level of community affluence, women’s education and employment and ethnic diversity. A higher mean/median score denotes a high level of community affluence, women’s education and employment and ethnic diversity. As presented in Table 1, the mean score for all the community characteristics ranged from 0.19 for ethnic diversity to 0.63 for community level of adult women’s employment. The majority of the adolescents in our sample resided in less affluent and less diverse communities. However, the level of women’s employment for pay in the communities appears to be high (0.631 ± 0.192).

**Table 1:** Descriptive characteristics of adolescents girls in the sample.

	Frequency Distribution	Percentages
Educational Attainment		
< Secondary	2,512	34.43%
Secondary +	4,937	65.57%
Place of Residence		
Urban	3,070	46.54%
Rural	4,379	53.46%
Region of Residence		
North Central	1,388	13.60%
North East	1,458	17.80%
North West	2,013	32.43%
South East	922	10.61%
South South	838	10.57%
South West	830	14.99%
Marital Status		
Ever Married	1,243	17.98%
Never Married	6,206	82.02
Ethnicity		
Yoruba	846	13.85%
Igbo	1,100	13.61%
Hausa	2,179	33.78%
Fulani	472	5.54%
Ijaw	203	1.77%
Others	2,649	31.46%
Religious Affiliation		
Christian	3,436	41.78%
Muslim	3,956	57.71%
Others	57	0.51%

**Table 2:** Distribution of adolescent girls in the sample by community characteristics.

	Median [min—max]	Mean ± Standard deviation
Community Affluence *(proportion of households in the highest wealth quintile)*	0.31 [0–1]	0.409 ± 0.378
Community Level of Women’s Education *(proportion of adult women with tertiary education)*	0.38 [0–1]	0.413 ± 0.335
Community Level of Women’s Employment *(proportion of adult women working for pay)*	0.65 [0–1]	0.631 ± 0.192
Ethnic Diversity	0.11 [0–1]	0.186 ± 0.202

### Survivor probabilities of sexual debut among adolescent girls

**Fig 1:**
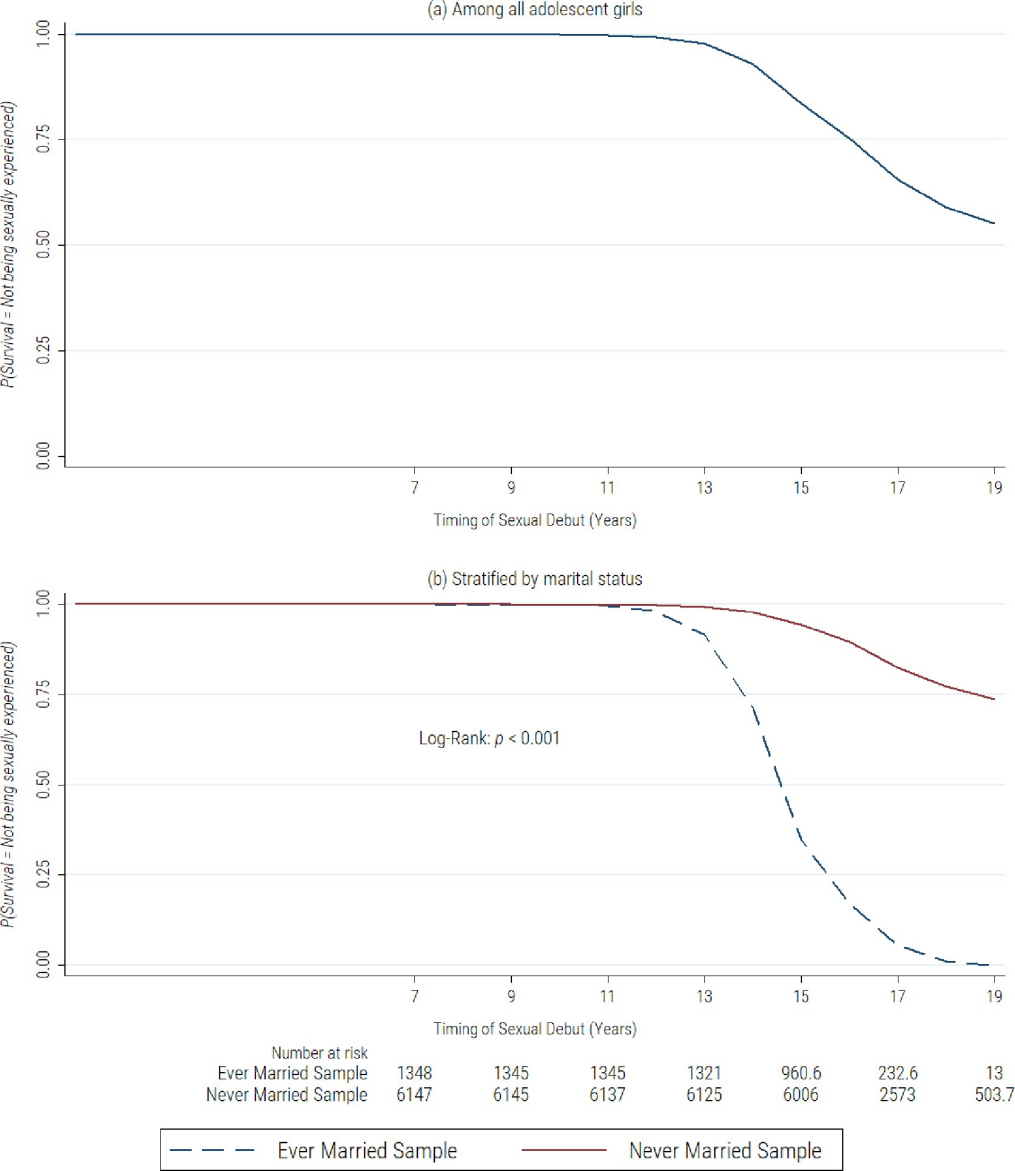
Survival probabilities of not being sexually experienced among adolescent girls in Nigeria.

Fig 1 presents weighted Kaplan–Meier survival estimates for the timing of sexual debut among adolescent girls in Nigeria. As presented in part a of Fig 1, we observed that the probability of not being sexually experienced was high in the younger ages, with steady declines from age 12. By 16 years, just about 75% of the adolescent girls are not sexually experienced, and by 19 years, nearly half of the adolescent girls would have been sexually experienced. Fig 1B presents the results of the Kaplan–Meier survival estimates stratified by marital status to assess the differential effect of marriage on the timing of sexual debut. We observed significant differences between married and unmarried adolescents in the probability of not being sexually experienced. The onset of sexual debut among married adolescents begins to decline at age 12 years with rapid declines before 17 years.

### Multivariate regression

Table 3 presents the multilevel cox-proportional hazard regression results with different model specifications. Model 1 presents the multilevel parametric survival model with only community-level characteristics. The result suggests that a higher community level of affluence and a higher level of women’s education within the community were negatively associated with the timing of sexual debut among adolescent girls in the sample. After adjusting for other relevant socioeconomic and demographic characteristics in Model 2, the result suggests that only the associations between the community-level of affluence remained significantly associated with the timing of sexual debut and in the same direction as Model 1. An increase in the proportion of households in the wealthiest quintile within the community is associated with a lower hazard [aHR: 0.43, 95% CI: 0.30–0.62] of sexual debut. A higher level of ethnic diversity was also associated with the timing of sexual debut after adjusting for other relevant characteristics. It emerged that higher levels of community diversity [aHR: 0.63, 95% CI: 0.42–0.94] are associated with a lower hazard of sexual debut. We also observed significant associations between marital status and the timing of sexual debut. Adolescent girls who have never been married have a lower hazard [aHR: 0.05, 95%CI: 0.04–0.06] of sexual debut than those who have ever been married.

**Table 3:** Multivariate multi-level mixed effect parametric analysis of timing of sexual debut among adolescents in Nigeria.

	Hazard Ratios [95% Confidence Interval]			
	Model I	Full Model	Sub-group analysis	
			Married Adolescents	Never Married Adolescents
Community Affluence *(proportion of households in the highest wealth quintile)*	0.23*** [0.16,0.32]	0.43*** [0.30,0.62]	0.87 [0.41,1.85]	0.40*** [0.27,0.60]
Community Level of Women’s Education *(proportion of adult women with tertiary education)*	0.56** [0.39,0.80]	1.64 [0.98,2.74]	0.52 [0.21,1.33]	2.45** [1.38,4.38]
Community Level of Women’s Employment *(proportion of adult women working for pay)*	0.84 [0.57,1.25]	0.97 [0.66,1.43]	0.82 [0.48,1.40]	1.85* [1.03,3.33]
Ethnic Diversity	0.84 [0.57,1.23]	0.63* [0.42,0.94]	0.68 [0.39,1.19]	0.56 [0.30,1.05]
Highest Level of Education				
< Secondary		Reference	Reference	Reference
Secondary +		0.60*** [0.49,0.72]	0.65* [0.46,0.92]	0.66** [0.51,0.87]
Place of Residence				
Urban		Reference	Reference	Reference
Rural		1.13 [0.94,1.35]	1.15 [0.83,1.60]	1.03 [0.83,1.27]
Marital Status				
Ever Married		Reference		
Never Married		0.05*** [0.04,0.06]		
Ethnicity				
Yoruba		Reference	Reference	Reference
Igbo		1.33 [0.65,2.69]	1.18 [0.47,3.00]	1.45 [0.69,3.05]
Hausa		0.99 [0.34,2.90]	1.47 [0.75,2.87]	0.63 [0.06,7.11]
Fulani		1.31 [0.48,3.59]	1.95 [0.95,3.99]	0.81 [0.21,3.13]
Ijaw		0.95 [0.40,2.26]	1.56 [0.55,4.46]	1.23 [0.50,3.03]
others		1.07 [0.48,2.36]	1.26 [0.66,2.41]	1.53 [0.68,3.48]
Region of Residence				
North central		Reference	Reference	Reference
North-East		0.59*** [0.45,0.79]	0.96 [0.66,1.42]	0.45*** [0.31,0.66]
North-West		0.60** [0.42,0.87]	1.08 [0.71,1.64]	0.17** [0.04,0.65]
South-East		0.57* [0.35,0.94]	1.12 [0.41,3.05]	0.49** [0.30,0.79]
South South		1.54** [1.16,2.03]	1.79 [0.88,3.63]	1.13 [0.86,1.48]
South-West		1.27 [0.68,2.37]	1.97** [1.19,3.29]	1.03 [0.55,1.91]
Religious Affiliation				
Christian		1.65*** [1.24,2.18]	1.66* [1.04,2.64]	1.42* [1.01,2.00]
Muslim		Reference	Reference	Reference
Others		0.82 [0.43,1.57]	0.85 [0.51,1.40]	0.93 [0.36,2.37]

* p<0.05

** p<0.01

*** p<0.001.

Sub-group analysis by marital status highlights differences in the patterns of associations between community-level characteristics and the timing of sexual debut among adolescent girls. We found no significant associations between the community-level characteristics and the timing of sexual debut among adolescent girls who have ever been married. On the other hand, we observed significant associations of community level of affluence and community level of women empowerment with the timing of sexual debut. Among the unmarried adolescent girls, we observed that an increase in the proportion of wealthy households within the community is associated with a lower hazard [aHR: 0.40, 95% CI: 0.27–0.60] of sexual debut. Higher levels of women empowerment evidenced in the level of women’s education [aHR: 2.45, 95% CI: 1.38–4.38] and employment [aHR: 1.85, 95% CI: 1.03–3.33], on the other hand, were significantly associated with a higher hazard of sexual debut.

Table 4 presents a summary of the neighbourhood variation and model comparison. As indicated in the table, the ICC in all the models is greater than 0.90, depicting that the correlation between log timing of sexual debut for adolescents and young women in the same neighbourhood is greater than 0.90. Furthermore, the PCV value of 0.381 in model 1 indicates that about 38% of the variation in the timing of sexual debut was attributable to only community-level factors. In comparison, about 69% of the variation in the timing of sexual debut is attributable to neighbourhood-level and individual-level factors.

**Table 4:** Summary of random effect and model comparison.

	Null model	Model I	Full Model	Sub-group analysis	
				Married Adolescents	Never Married Adolescents
Measures of variation					
Neighbourhood Variance	1.34 [1.14, 1.57]	0.83 [0.70–0.99]	0.42 [0.33, 0.54]	0.50 [0.36, 0.68]	0.50 [0.30, 0.83]
ICC	0.990 [0.988, 0.992	0.984 [0.981, 0.988]	0.976 [0.969, 0.983]	0.985 [0.979, 0.991]	0.972 [0.958, 0.987]
PCV	Reference	0.381	0.687		
Model fit					
AIC	2050.07	1730.9	-655.96	-2679.41	1699.62
BIC	2070.82	1779.31	-503.81	-2576.9	1841.02
Adolescent girls	7449	7449	7449	1243	6206
Households	6505	6505	6505	1228	5323
Neighbourhoods	1352	1352	1352	520	1291

ICC = Interclass Correlation, PCV = Proportional Change in Variance, AIC = Akaike Information Criterion, BIC = Bayesian Information Criterion.

## Discussion

Most sexuality research, especially studies of young females in sub-Saharan Africa, emphasize individual characteristics associated with the timing of sexual debut. As a result, we know comparatively little about contextual determinants of timing for first sex among young adults in Nigeria. This study contributed to the literature by examining the association between community characteristics and timing of sexual debut among young adults in Nigeria. An awareness of such relationships may be essential to design sustainable policies that account for different contexts to address sexually transmitted infections and unplanned pregnancies among young adults. Furthermore, knowledge about the timing of sexual debut in different contexts will allow program planners to provide age-appropriate sexuality education. Suppose the program planners believe that young adults initiate sex at later ages based on norms regarding sexual behaviours in different communities in Nigeria; this may exclude the younger cohort of young adults in policy and legislation.

Our results align with our first hypothesis, which supports Billy, Brewster [36] seminal work on adolescent girls’ community context and sexual behaviour and other studies on older women that focused on multiple concurrent sexual partners [43]. The protective role of wealth at the community level for the delay in the timing of sexual debut was evident in our results. We expected this because wealth at the community level can make available resources that help promote the required sexual education content for young adults. In Nigeria, it is also possible that communities with a high proportion of adult women residing in wealthy households are less likely to adhere to traditional norms, which may help them better inform young adults on strategies to delay sexual debut. Poverty is a risk factor for transactional sex among adolescents, so young adults from communities with a high proportion of adult women in wealthy households may be protected from having to exchange sex for gifts. Nigeria is a patriarchal society, and the gender inequality perpetrated in several communities that aggravates adolescent vulnerability to transactional sex may not exist in communities with a high proportion of rich adult women. This possibility may delay the timing of sexual activity among young adults.

We also found support for the hypothesized association between community level measures of women empowerment and the timing of sexual debut among young adults, albeit in an unexpected direction. Precisely, we observed that a higher proportion of highly educated adult women and women working for pay was significantly associated with a higher hazard of sexual debut during adolescence. Our results do not support our other propositions. Nonetheless, a multi-country study of adolescents in sub-Saharan African countries also found that females living in communities with a high proportion of educated adults were less likely to delay sex [31]. These are unexpected results, and we find a little explanation for these results in the literature, which we propose should be a frontier for future research.

Another reason for our findings could be our measure of community education, which focuses on educational attainment, not the type of educational institution attended or the quality of education received, which may be more protective in the lives of adolescents. In addition, several studies [34] have demonstrated the protective effect of community employment on youth sexual behaviour. However, we have contrary results. It is possible that highly educated and employed women in these communities spend more time at work and less time supervising adolescents. Reduced parental monitoring may result in earlier timing to sexual debut among young adults [44].

It is possible that women in the communities are educated but lack sexual education or do not know how to educate adolescents, which may explain our results. A commentary by Shtarkshall, Santelli [45] has suggested that program planners must look beyond education, a structured way of imparting knowledge and focusing on sexual literacy.

We find no association between community diversity and youth sexual behaviour. This result is also unexpected as we believed ethnicity would represent the different norms of women in the community regarding sex which may ultimately influence youth sexual behaviour.

Although our study contributes to the literature in meaningful ways, it is not without limitations. The first is that although we excluded women exposed to sexual activity after moving, this exclusion limits our sample to those who have not left their communities. Given that migration might influence sexual initiation or marriage, the unique characteristics of movers may differ from those included in this analysis. As a result, our findings may not generalize beyond the population group studied in this work.

Furthermore, all socioeconomic characteristics and community variables were measured during data collection. We expect that they may not have changed significantly since sexual initiation, and as a result, the findings should be interpreted with caution. Another limitation of this study is the cross-sectional nature of the data. Although, we identified multiple links between the timing of sexual debut among adolescent girls, we are not able to assess the extent to which these are causal relationship or stems from unobserved heterogeneity. Future research needs longitudinal data at the individual and community level. Modelling the interactions between these levels of influence at different time points may also be helpful. Such studies are more complex and expensive than most research that has been conducted to date and require prioritization by international funding bodies. However, if we are to develop genuinely effective policies, strategies, and programs to support the healthy development of youth, such research is essential.

## Conclusion

We find mixed support for the association between community characteristics and the onset of sexual debut among young adults in Nigeria. We found that high community affluence was significantly associated with a lower hazard of premarital sexual debut. On the contrary, a higher proportion of adult women with tertiary education and women working for pay within the community were significantly associated with a higher hazard of sexual debut among young adults. This finding highlights the significance of considering specific community contexts in designing policies for youth and looking beyond education, and focusing more on sexual education.

A significant contribution of this study is examining the relationship between community variables and the timing of sexual debut among young adults. Our unexpected results also call for more research among young adults in Nigeria. The strength of our results is the sampling strategy and the sample size of the dataset.
